# Surgery

**Published:** 1838-04

**Authors:** 


### SURGERY.

*Report of Surgical Cases, treated in the Glasgow Royal Infirmary, during the Years 1836-7.* By WM. DAVIDSON, M.D., Surgeon to the Glasgow Royal Infirmary.

THIS is a long and very valuable Report; but, as it consists, for the most part, of histories of single cases, we are prevented from laying a general analysis of it before our readers: it will, however, well repay a careful perusal. The cases are graphically described, and the remarks on them highly judicious and instructive. We can only find room for one or two brief extracts.

*Hydrocele.* Two cases are related, which were treated by acupuncture, but without permanent benefit. On this subject Dr. Davidson remarks: "Were it in general ultimately successful, it would be the most simple, and at the same time the most extraordinary, operation that is recorded in surgery. Even upon the supposition that it is only a palliative or substitute for the common plan of tapping with a trocar, the discoverer is entitled to much credit; for a puncture with a sewing-needle is attended with scarcely any pain, and the most timid of men would submit to it without apprehension. The results arising from the treatment of two cases are not enough for drawing any certain conclusions; but certainly they tend to establish this point, that no particular change is effected upon the internal coat of the tunica vaginalis, and that the fluid reaccumulates as after ordinary tapping with a trocar. . . .

"It may be stated that the cases above mentioned were not well adapted for the plan; for in both the testicles were enlarged. This certainly is an objection to the success of acupuncture, as well as to every other plan for a radical cure of hydrocele; but a little enlargement of this organ is a very common occurrence in this disease, and in the first case detailed the enlargement had almost completely subsided before he left the house. It appears to me that this operation is not likely to supersede the radical cure by injection, but that as a palliative it ought generally to be preferred to the use of the trocar; at the same time, I am of opinion that a frequent and long-continued use of the needle may in some cases effect a radical cure; and this view is supported by the fact, that after acupuncture has been repeated several times, the re-accumulation of fluid goes on less rapidly than after the first or second operation."

*Erysipelas.* This disease infested some of the wards in the infirmary notwithstanding the greatest care to avoid all the probable causes of it, and its attacks rendered abortive several operations and proved not unfrequently fatal. Some interesting cases are related. We quote the following, not, however, as a specimen of the disease, but as exhibiting a novel method of evacuating the contents of abscesses.

"In the latter end of January, 1837, a lad, about sixteen years of age, who was admitted for *necrosis* of the leg and arm, and where a circular piece of necrosed bone, about two inches and a half long, had been extracted from the tibia, was seized with the disease in about eight days after the operation. The disease made its first appearance in the knee, and spread rapidly upwards to the groin; pulse 130, feeble, and he had frequent vomiting. An abscess took place on the dorsum of the foot, which was opened, and the knee-joint became much swollen on both sides of the patella, attended with a throbbing pain. It was evident that purulent matter had collected in the joint; but as making a free opening into it would have most probably increased the constitutional irritation, the following method of treatment was adopted. A grooved needle, about a line in diameter, was introduced into the most prominent part of the swelling, on the inner side of the joint, which gave exit to a sero-purulent fluid. A cupping-glass was now applied over the puncture, and about three ounces of the same fluid were evacuated. The wound was found completely closed next day, and the same operation was repeated every successive day, for a few days, with the evacuation of from one to two ounces of fluid each time. He left the hospital in about a week afterwards, his limb being nearly restored to its former state, with the exception of the knee, which was still somewhat swollen.

"This plan of using the grooved needle and cupping-glass might be advantageously employed, not only in evacuating collections of matter in joints, but also in some chronic abscesses, where high constitutional irritation is apt to follow a free incision."

The following extract exhibits the general mode of treatment adopted, and its results:

"In the general treatment of erysipelas, the antiphlogistic plan was only employed in a few cases in the commencement of the disease; for it was found that symptoms of debility in general appeared pretty early, more especially if diarrhœa was a concomitant, which was not unfrequent. The tonic plan was, therefore, found the most successful, and it consisted of wine, sulphate of quinine, light nourishing diet suited to the state of the digestive organs, laxatives or laxative enemata, and occasionally opiates at bed-time. The external treatment consisted of leeches, punctures, incisions, mercurial ointment, nitrate of silver applied in the form of a weak solution to the whole erysipelatous surfaces, or applied in the solid state in the form of a circle, with the intention of insulating the disease. Leeches and punctures were not found so beneficial as incisions; and the latter were generally practised, and made in various parts of the region affected, to the extent of from one inch and a half to two inches and a half in length, through the skin and cellular texture. In the slighter cases, where the disease appeared to be superficial, mercurial ointment, and a solution of nitrate of silver, consisting of ten grains to an ounce of water, were applied; but the latter was found the most efficacious of the two; and generally, on the day following its application, the swelling and redness were much diminished. The solid nitrate of silver succeeded, in the great majority of cases, in preventing the spreading of erysipelas; and the following points require to be attended to, in order to ensure success. 1st. It must be applied to a sound part of the integuments, viz. a part where there is no swelling or redness; but as near as possible, so as to avoid this. 2d. The inflamed surface must be completely encircled by the caustic line. This may be effected in the following way. Take a pretty large hair-brush and moisten thoroughly with water the part that has been selected to the breadth of about an inch; then rub a cylinder of lunar caustic very freely over this moistened portion of the skin. Distinct vesication over the whole surface to which the caustic has been applied should be produced; for if this does not follow, the disease may extend beyond the line. And this is perhaps the reason why a saturated solution (consisting of equal parts of the salt and water) is not so certain as the solid caustic, for erysipelas seems to extend its boundaries by creeping along the cutaneous surface, before it affects the cellular tissue; hence if its progress over the integuments can be checked, its extension in the textures below will at the same time be prevented. In general, after the caustic has been thus applied, the inflamed integuments in the immediate vicinity of it become partially shrunk and puckered; but the state of the previously affected parts appears to be uninfluenced by it, and they proceed to resolution or suppuration, according to the nature of the case. Many cases could be quoted from the journals of the house, besides those already noticed, where this practice was adopted, in proof of the general efficacy of this mode of insulating erysipelatous inflammation; but their introduction would render this report too long." *Edinburgh Journal. Jan.* 1838.

---

### *Singular Case in which a Steel Fork was Extracted from the Back.*
### By DAVID BURNES, M.D.

ROBERT SYMS, aged twenty-three, was entered on the sick list of H. M. ship *Belvidera*, about the middle of June, 1831, complaining of pain at the inferior angle of the right scapula, close to the base of which was a small phlegmon, as I then considered it, in the early stage of suppuration. On the 19th of June, I opened "the boil," and ordered poultices to be applied, thinking it would heal kindly in a few days. On the 23d, however, on probing the wound, I felt what I first thought was the edge of the scapula, but, on more minute examination, something black and shining was seen in the wound. On the 24th, it being evident that there was some foreign body in the wound, the opening was enlarged directly upwards, and a piece of steel, about the thickness of a common ram-rod, presented itself, but resisted strongly any efforts to extract it. Being unwilling to put him to further pain, while there was a chance of its coming away by poulticing, and pulling it with the forceps daily, this gentler course was agreed on in preference to making a further enlargement of the wound. Being questioned as to the nature of the piece of steel, he expressed himself as much astonished as we were at its presence, and said he should not have known it had we not told him, and had he not felt pain from our pulling it with the forceps. He had never been in action, having been only two years in the king's service, nor did he recollect having received any wound by which anything of the kind could have been introduced. About two inches below the opening made on the 19th, we observed a small white speck, or mark, rather resembling the mark left many years after vaccination, than a cicatrix of a wound. This was the only vestige of anything like a wound that we could detect in his back.

July 2. The poulticing has been continued, and there is now a free discharge from the wound; the steel has been pulled daily by the forceps, and admits now of farther motion, especially laterally, but is yet forcibly retained at its upper part; its direction is nearly parallel with the base of the scapula, close to which it lies, and in its course upwards it seems to incline deep into the substance of the muscles. About an inch of it can be seen when the integuments are retracted. He is averse to further measures; has no pain except from the use of the forceps. Continue the poultices.

16th. Though the poulticing has been continued, and the steel pulled daily, there is no material alteration since last report, further than that the steel may be moved more freely in every direction, except when pulled directly downwards, when it seems to be retained as forcibly as at first; the probe can be introduced into the wound, upwards and inwards, nearly four inches, and can with some difficulty be made to move round the steel; but no information as to its size or shape can be gained from this mode of examination. It occurred to me, at this time, that it was a hook, and that it might be retained by catching on one of the ribs. Having no pain except from the pulling, and being still averse to the use of the knife, the same treatment was pursued.

August 5. The foreign body having become very little loosened, and now causing more pain on its being moved, I made a deep incision of about three inches in length over its course upwards, using it as a director, when it was easily extracted, and found to be a common kitchen fork, broken off close to its handle, and with one of its two prongs wanting about an inch from its point; it was blackened, and, in some degree, rusted. It seemed to have been retained by a bridle of muscular fibres embracing its shoulders, for it was immediately liberated when the part was divided by the knife.

The wound was dressed simply, and healed so soon that in ten days the man was doing duty in the boats and aloft.

Strange as it may seem, even after its extraction, the man persisted in adhering to his original statement of his being ignorant, how and when it had been introduced; and during the two months I remained in the ship, I was not able to gain further information on the matter.

The patient continued to serve in the *Belvidera* till December, 1833, when he joined H. M. ship *Blonde*, going to South America. Being anxious to trace his future history, in the hope of obtaining some clue as to the introduction of the fork, I was enabled, through the kindness of Sir William Burnett, the Physician-General of the Navy, on the arrival of the *Blonde*, at Portsmouth, about a month ago, to communicate with him by letter. The result was, that he came up to London, and, on the 18th of November, called upon me to show himself. He then stated that, about eighteen months ago, while washing himself, he felt a small, hard body on the *left* side of the neck, which he was inclined to believe was part of the fork. On examining the part I had no doubt myself of its being the portion of the broken prong, and which I asked permission to extract. He readily assented.

On the 20th, in the presence of Mr. C. Smith, surgeon, I made an incision over it (its position being just behind the middle part of the posterior edge of the sterno-cleido mastoideus muscle, where it is crossed by the external jugular vein, when it was easily removed, and proved to be the prong, which had the same bronzed appearance as the fork itself, and was coated with rust at its fractured end. It does not exactly join with the fork, and I am inclined to think some very minute splinters may have been broken from it when fractured, or some chemical action, while in the body, may have corroded it.

It is singular that he had never suffered pain from it, although it has crossed from the *right* side of the back to the *left* side of the neck. I was only induced to extract it from its superficial position, and the singularity of the history, yet it is possible it might, in time, have advanced still further, and have injured the carotid artery, or trachea. Though cross-questioned by all who saw him, he still repeats his former story of being innocent as to the introduction of the fork.

*Lancet. December* 2, 1837.

---

*Case of Rupture of an Aneurism of the Common Carotid, and Ligature of that Artery near its Origin from the Innominata.* By T. Argyll Robertson, M.D., F.R.S.E., Lecturer on Surgery in Edinburgh.

[The following case, as the author observes, is deserving record as being the only one in which a ligature has been applied to one of the larger arteries of the body, nearest to their common origin from the heart, with perfect ultimate success.]

Major ——, the subject of the case, is now in his fifty-second year. In April, 1836, while hunting, his horse, when at full speed, put its foot into a rabbit hole, by which both it and its rider were brought to the ground with great violence. Major —— received a severe wound over the left parietal bone, which bled profusely, and he remained for a short time stunned, and in a state of insensibility. From that period he suffered from stiffness and pain in the right side of the neck, resembling what is usually termed a crick, accompanied by shooting pains over the whole right side of the head: occasional attacks of giddiness. . . . About the middle of January last he first discovered a swelling on the right side of the neck, accompanied by enlarged tonsils, slight sore throat, and some difficulty in swallowing. The swelling in the neck was supposed to be simply an enlarged gland, and did not attract particular notice. . . . On the 20th of March, without any premonitory symptoms, at ten o'clock, P.M., a sudden gush of blood took place from the mouth; it was discharged in gulps or mouthfuls in rapid succession; it ceased spontaneously after half an ordinary wash-hand basinful of blood had been lost; he retired to bed, and slept very soundly during the whole night. The following morning he rose at eight o'clock, but had scarcely reached his dressing-room when the hemorrhage returned, and, to use his own expression, the blood literally poured from his mouth; he soon fainted and fell upon the floor, breaking a foot pail in the fall; about fifty ounces of blood were at this time discharged by the mouth, and a considerable quantity must have passed into the stomach, as the stools afterwards consisted almost entirely of coagulated and grumous blood. By the two hemorrhages he must have lost upwards of one hundred ounces of blood. The bleeding now ceased, and he rallied a little and procured assistance . . . I reached Major ——'s seat about midnight, and found him perfectly composed and tranquil, with the pulse scarcely perceptible at the wrist, and continuing to beat about forty-five strokes in the minute. On examining the neck I found a tumour extending from near the angle of the jaw to within one inch of the sternum, and projecting laterally to about three inches. Its surface was smooth, equal, and rounded, and a very obscure pulsation could be detected. Judging from the state of the circulation, and from there not having been the slightest return of bleeding since eight A.M., that there was not immediate danger, I thought proper to postpone attempting to secure the carotid below the seat of the aneurismal swelling, (evidently the only surgical resource that was left me,) in order that I might have the benefit of daylight for the operation.

In consequence of the aneurism being seated so low down in the neck, the external incision was limited to little more than an inch, following the course of the sterno-mastoid from the sternum upwards. On dividing transversely the sterno-thyroid and a few fibres of the sterno-hyoid muscles, a narrow projection of the aneurismal tumour, passing between the artery and trachea, was brought into view; so narrow that I at first supposed it to be the artery somewhat dilated, and passed the aneurism needle round it. It was, however, about double the size of the artery, and its coats were thinner than natural. On examining the parts more minutely, I discovered the carotid displaced laterally by this prolongation of the sac. The pulsations were feeble, though perfectly distinct; the situation at which it was exposed was within a finger's breadth of its origin from the innominata, and when the finger was applied to this point the carotid was felt pulsating on its palmar, the innominata on its lateral surface. Immediately above this point the vessel swelled out into the aneurismal tumour. The ligature was therefore applied within half an inch of the origin of the artery. During the performance of the operation neither vein nor nerves were seen. The operation itself was necessarily tedious and difficult, in consequence of the limited extent of the external incision, the deep situation and unnatural displacement of the artery, and the importance of the organs by which it is surrounded. At one time I thought it would have been necessary to have divided the sternal attachment of the sterno-mastoid, but this was avoided by relaxing the muscle and drawing it outwards. The vessel was no further separated from its attachments than was necessary for the simple passage of the aneurism needle. On tightening the ligature all pulsation ceased in the tumour, and it was reduced nearly a third in bulk. No peculiar sensations were experienced by the patient, who bore the operation with the greatest possible fortitude. He was placed in bed, with the head considerably elevated to relax the parts, the lips of the wound having been previously brought together by suture, and supported by a strip of adhesive plaster. Strict antiphlogistic regimen and perfect rest were enjoined. The bowels were regulated by enemata to avoid any risk of sickness, vomiting, or hypercatharsis from the exhibition of cathartics. The wound healed by granulation; at first the discharge was thin and slightly tinged with blood, but gradually it assumed the characters of healthy pus.

On the seventeenth day the ligature separated, and the wound speedily healed. The aneurismal tumour rapidly disappeared, and now no trace of it whatever can be discovered. From the period at which the ligature was applied up to the completion of the cure not an untoward symptom appeared. On the second day after the operation the pulsation in the branches of the external carotid was distinct. During the third week a few drops of blood were discharged from the right nostril, accompanied by a little irritation giving rise to a great desire to sneeze, probably depending on the new arrangement of the circulation. At the present time, 28th September, six months from the date of the operation, Major —— is in the enjoyment of the most perfect health. *Dublin Journal. January,* 1838.

---

### *Observations on the Pathology of Staphyloma.*
By T. Wharton Jones, Esq. London.

This is a valuable paper, exhibiting the author's familiarity with the diseases of the eye and the nice operations indicated in the removal of so many of them. We regret that our space permits us to lay only a portion of the paper before our readers.

"The only mode of formation of staphyloma which I could ever trace is the following:

"If, in scrofulous, catarrhal, or catarrho-rheumatic ophthalmia, there be a penetrating ulcer of the cornea, the aqueous humour escapes, the iris falls forward into contact with the cornea, and a small part of it is perhaps prolapsed through the ulcerated opening. The progress of the ulceration being stopped by the yielding of the inflammation, the prolapsed portion of iris, and the ulcerated part of the cornea, are involved in one cicatrice. The opening in the cornea being thus closed, the aqueous humour again collects, and the anterior chamber is restored, though somewhat diminished, in consequence of the partial adhesion between the iris and cornea (*synechia anterior.*) There is no prominent distension on the front of the eye in this case, because, as the inflammation subsides, the small protruded portion of iris shrinks and flattens; but if the destruction of the cornea has gone on farther, either by ulceration or the giving way of an onyx, and considerably more of the iris has protruded, the prolapsed portion of the iris does not shrink when the inflammation begins to abate, as in the former case, but remains, and forms a projection at one part of the cornea, generally the lower or lateral. This projection is at first merely a bag of the iris distended by the aqueous humour; but by and by its exposed surface becomes covered by an opaque firm tissue, of the nature of the tissue of cicatrices, and this tissue is incorporated at the base of the tumour with the sound cornea. The projection, the mode of origin I have just described, is a partial staphyloma; it is not a distension of the cornea itself, but a protruded portion of the iris covered by a new tissue, intended to supply the loss of substance which the cornea has sustained. The mode of origin of a total staphyloma is essentially the same, but differs only in degree. The whole or greater part of the cornea being destroyed, as occurs in gonorrhœal, purulent, and very often in variolous ophthalmia, as also that of new-born infants, the whole iris falls forward, the pupil becomes closed, and the aqueous humour being thus allowed to accumulate in the posterior chamber, the iris is kept distended in the form of a tumour on the front of the eye. Its surface gradually gets covered with an opaque cicatrice-like tissue, or pseudo-cornea, which assumes a greater or less degree of thickness, and a total staphyloma is the result. Sometimes the central part of the cornea only is destroyed, a ring of the circumference still remaining; the staphylomatous projection has then the form of a small globe stuck on the front of a larger.

"Founding my reasonings on those views, I conceived that the supply of aqueous humour in the still-existing posterior chamber was what kept up the distension of the iris, and the consequent moulding of the pseudo-cornea on its surface in the form of a round prominence on the front of the eye. If, therefore, it was natural to infer the source of the aqueous humour could be destroyed, we should not have the development of the staphylomatous projection, or, if already formed, it would disappear. For this purpose puncturing of the tumour is not found to answer well. To break in upon the integrity of the posterior chamber, I conceived the simplest and most effectual plan would be to extract the lens, an operation which I put into practice in the following case.

"A young man, about twenty-two years old, came to me labouring under the effects of severe purulent ophthalmia of both eyes. In the right eye I found the cornea destroyed, and the iris protruding and distended with aqueous humour, the pupil being closed. The left eye had also suffered very much; there was penetrating ulcer, prolapsus iridis, and consequently considerable distortion and contraction of the pupil. Both eyes were still affected with the inflammation, and it was very doubtful whether the left eye could be prevented from getting worse, especially as it was evidently kept in a state of additional irritation from the presence of the staphyloma in the right. By an incision with a Beer's cataract knife in the protruding and distended iris, the lens was extracted. Severe re-action followed; less perhaps in consequence of the operation, than in consequence of the patient not having been in a situation to take proper care of himself. The iris did not again become distended; on the contrary the eye shrunk, and irritation being thus removed, the left eye progressively improved, as far as the organic changes it had already undergone allowed, and further than there had been previously reason to hope for, as vision was preserved sufficient to enable him to resume his employment as a porter."

"The conclusions which I draw from my observations are the following:

"That the iris and cornea do not unite surface to surface, and if they unite at all it is only partially, and that in consequence of penetrating ulceration of the latter, and prolapse of the former.

"That the tissue composing a staphyloma is not degenerated and opaque cornea, but a new tissue, of the nature of the tissue of cicatrice, developed on the anterior surface of the iris exposed by the destruction of the cornea itself."

*Med. Gazette. February* 24, 1838.

---

*Case of Fatal Convulsion during the Injection of Nævus.* By T. PAGET, Esq., and F. FULLAGAR, Esq. Leicester.

"THE following impressive case," say the relators, "seems due to the medical world, as an instance of the distressing casualties which beset the hopes of our profession." No blame whatever can attach to the operators; while every one must sympathise with them, and admire the candour with which the facts are stated.

"The nævus was situated over the angle of the right maxilla, in a healthy and remarkably precocious child, nearly two years old, and had increased from a slight speck at birth to the size of half a small orange. It was principally subcutaneous, but had a coloured portion in its centre, which seemed covered by cuticle only. On the 19th of October the tumour was pierced with a lancet used for the nasal duct, and injected by the transfusion syringe; the mixture used being nitric æther, with one fifteenth of nitric acid. No decided effect was produced, so that either too little of the injection had entered or the mixture was too slightly stimulating, and in about a week the operation was repeated; the proportion of nitric acid being increased to one-tenth. No more effect, however, followed this than the former operation. On the 15th of November the injection was again had recourse to: using, instead of the nitric acid and æther, the liquor ammoniæ, weakened so that the nose could remain applied to the bottle. The perforation was this time made from the opposite side, and more attention being paid to the compression of the tumour, the piston was felt to descend more, and the tumour appeared more injected. At this instant the poor child suddenly ceased its crying, and at the end of a slight convulsion, continuing not a minute, lay upon the table a corpse.

"As to the cause of this melancholy result, the first hurried impression at the conclusion of this harassing scene was, that the attack was merely coincident with the operation; that, in fact, it was one of those sudden and fatal succussions of the nervous system which now and then carry off children, apparently without reason."

"Yet though it is impossible to deny that such was the secret of this appalling case, and the harassed feelings cling to the supposition, it seems at least as impossible to deny, that some of the stimulating fluid was forced into the divided veins, thence carried along the external jugular and subclavian to the heart, and thus produced the death; or that a strong irritant, applied to the branches of the seventh and third nerves, is as likely to excite a fatal succession of the whole nervous system, as those doubtful yet accredited agents, dentition and gastric sordes. Either of these occurrences would be decisive against the operation, and their possibility demands the publication of the case. No post-mortem examination was allowed. However unlikely it is that air should be forced any where but into the cellular tissue of the nævus, it may be well to state, that it was impossible in this case, as the syringe was first filled with particular care, and afterwards held nozzle upwards, to insure the perfect filling of its pipe, and the escape of any air, if possibly any were left in.

*Med. Gazette. December* 30, 1837.

